# Model based heritability scores for high-throughput sequencing data

**DOI:** 10.1186/s12859-017-1539-6

**Published:** 2017-03-02

**Authors:** Pratyaydipta Rudra, W. Jenny Shi, Brian Vestal, Pamela H. Russell, Aaron Odell, Robin D. Dowell, Richard A. Radcliffe, Laura M. Saba, Katerina Kechris

**Affiliations:** 1 Department of Biostatistics and Informatics, University of Colorado School of Public Health, Aurora, CO 80045 USA; 2 Computational Bioscience Program, University of Colorado School of Medicine, Aurora, CO 80045 USA; 3 Department of Biology, University of Oregon, Eugene, OR USA; 4 Department of Molecular, Cellular and Developmental Biology, University of Colorado at Boulder, Boulder, CO 80303 USA; 5 BioFrontiers Institute, Boulder, CO 80303 USA; 6 Department of Pharmaceutical Sciences, University of Colorado Skaggs School of Pharmaceutical Sciences, Aurora, CO 80045 USA

**Keywords:** Heritability, RNAseq, Recombinant inbred panel, Negative binomial mixed model, Compound Poisson mixed model, Variance partition coefficient

## Abstract

**Background:**

Heritability of a phenotypic or molecular trait measures the proportion of variance that is attributable to genotypic variance. It is an important concept in breeding and genetics. Few methods are available for calculating heritability for traits derived from high-throughput sequencing.

**Results:**

We propose several statistical models and different methods to compute and test a heritability measure for such data based on linear and generalized linear mixed effects models. We also provide methodology for hypothesis testing and interval estimation. Our analyses show that, among the methods, the negative binomial mixed model (NB-fit), compound Poisson mixed model (CP-fit), and the variance stabilizing transformed linear mixed model (VST) outperform the voom-transformed linear mixed model (voom). NB-fit and VST appear to be more robust than CP-fit for estimating and testing the heritability scores, while NB-fit is the most computationally expensive. CP-fit performed best in terms of the coverage of the confidence intervals. In addition, we applied the methods to both microRNA (miRNA) and messenger RNA (mRNA) sequencing datasets from a recombinant inbred mouse panel. We show that miRNA and mRNA expression can be a highly heritable molecular trait in mouse, and that some top heritable features coincide with expression quantitative trait loci.

**Conclusions:**

The models and methods we investigated in this manuscript is applicable and extendable to sequencing experiments where some biological replicates are available and the environmental variation is properly controlled. The CP-fit approach for assessing heritability was implemented for the first time to our knowledge. All the methods presented, as well as the generation of simulated sequencing data under either negative binomial or compound Poisson mixed models, are provided in the R package **HeritSeq**.

**Electronic supplementary material:**

The online version of this article (doi:10.1186/s12859-017-1539-6) contains supplementary material, which is available to authorized users.

## Background

Heritability is an important concept in genetics and provides a quantitative measure for the proportion of trait variation that is attributed to genetic variation. It is used in a variety of fields including psychology, behavioral genetics, and breeding [1, 2]. For example, the heritability of cow milk yield is crucial for cattle breeders. The heritability of the desirable trait, either phenotypic or molecular, can then be used to predict the genetic merit of a cow and help design an appropriate breeding program [3]. Other uses of heritability are relevant to the study of disease. If a pattern of inheritance for a disease in a family has been discovered, it can lead one to hypothesize that one or more molecular traits are in part responsible for the development of the disease [4, 5]. Heritability analysis may then be applied to evaluate the likelihood of an offspring inheriting the molecular traits and/or the probability of presenting the symptoms of the disease. This usage can be extended to personalized medicine. A large list of traits might be relevant to a disorder, and as a quantitative measure, heritability provides a way to rank the traits and helps to prioritize candidates for further investigation [6].

Heritability may be measured for “intermediate” or molecular traits for a physiological or disease state. Gene expression has been well studied as an intermediate trait in genomics [7–9]. The study of expression quantitative trait loci (eQTL) and heritability of gene expression is relevant for understanding the basis of complex traits [10]. However, most of the expression trait methodologies are based on microarray technology and assume the data to be Gaussian [11]. The rise of high throughput sequencing (HTS) technology demands the development of new methods as it produces data that are highly non-Gaussian. Such technology has several advantages over microarrays and is now favored by most researchers [12]. High throughput sequencing studies of RNA (RNA-Seq) result in sequence reads that are mapped to a gene or other genomic feature, and the mapping procedure produces count data for each feature. The mean-variance relationship in the data is usually different from that of a Gaussian distribution [13]. Sun [14] proposed a method based on negative binomial regression to find eQTL in RNA-Seq data. However, methodologies to estimate heritability scores for such data are lacking. We propose several statistical models and different methods to estimate heritability for high throughput sequencing data based on linear and generalized linear mixed effects models.

In animals and plants, panels of Recombinant Inbred (RI) strains are an excellent resource for systems genetics studies. RI strains have been used in genetic mapping for over four decades [15, 16]. Details on the origin and history of RI strains can be found in [17]. Since RI panels allow replicated samples with nearly homogeneous genetic information per strain under a controlled environment, they have the advantage of reproducible genotypes and the ability to separate genetic variability from environmental variability. Therefore, an RI panel with an adequate number of replicates and strains facilitates analysis of complex traits and heritability. In this work, we analyzed microRNA (miRNA) and messenger RNA (mRNA) sequencing data from a large RI mouse panel that have been bred from reciprocal crosses between the Inbred Long Sleep (ILS) and Inbred Short Sleep (ISS) strains, called the ILSXISS (LXS) panel. The original long-sleep and short-sleep lines were selectively bred for a long or short duration of loss of righting reflex due to ethanol (i.e., sleep time) [18, 19]. Although the expression data from the LXS panel motivated this work, the methods described in this paper are applicable to any HTS data with biological replicates and properly controlled environmental variation and population structure.

We present four methods for estimating heritability that account for the challenges posed by count and possibly zero-inflated data. Since many of these alternatives are non-linear models, standard methods do not apply and we introduce the usage of the Variance Partition Coefficient (VPC). We also propose a statistical test for evaluating whether the heritability is greater than zero and a method for defining confidence intervals. Simulations and the motivating data including miRNA and mRNA expression from the LXS panel are used to compare and test the methods. We give recommendations about the performance of the heritability methods under different situations. Finally, our methods are provided as an R package HeritSeq.

## Methods

### Definition of heritability

Heritability of a genetic or phenotypic (*P*) trait is the proportion of total variance (*V*_*P*_) that is attributable to genotypic (*G*) variance (*V*_*G*_). The classical approach assumes that the total variance *V*_*P*_ can be partitioned into *V*_*G*_, the variance attributable to genotypes, and *V*_*E*_, the remaining variability, which is also known as variance due to environment (*E*). In other words, *V*_*P*_=*V*_*G*_+*V*_*E*_. The (broad sense) heritability, *H* ^2^=*V*_*G*_/*V*_*P*_, is “the proportion of phenotypic differences due to all sources of genetic variance” [20].

The two most common approaches for estimating heritability differ based on the population and sample being studied. One is based on analysis of correlations and regression, first developed by Sewall Wright then popularized by Ching Chun Li and Jay Laurence Lush [21–24]. Traditionally, this approach estimates heritability from simple, often balanced designs, and computes the correlation of offspring and parental traits, the correlation of full or half siblings, or the difference in the correlation of monozygotic and dizygotic twin pairs. The second approach is based on the analysis of variance (ANOVA) in breeding studies, using intraclass correlation among relatives and was originally developed by Ronald A. Fisher [24, 25]. Given the nature of RI panels, our methods follow the latter approach and focus on estimation of variance components.

### Heritability for linear mixed models: intra-class correlation

A common approach to estimate heritability is to apply a random effects analysis of variance model and use the intra-class correlation (ICC) [26]. Such a model usually involves a fixed intercept, a random effect term to explain the effect of genetic background (which can be represented by different strains) and an error term that explains all other non-genetic variation. A simple model can be

1 $$  y=\alpha+b_{s}+\epsilon, \; b_{s} \sim N\left(0,\sigma^{2}_{g}\right), \; \epsilon \sim N\left(0,\sigma^{2}_{\epsilon}\right),  $$

where *y* is the trait, *α* is the fixed intercept, *b*_*s*_ is the random effect due to strain *s* and *ε* is the random error. $\sigma ^{2}_{g}=Var(b_{s})$ is the variance due to genotype and $\sigma ^{2}_{\epsilon }=Var(\epsilon)$ is the error variance.

For the above linear mixed model, the variance components are additive so the total variance can be written as

2 $$  Var(y)=\sigma^{2}_{g} + \sigma^{2}_{\epsilon}.  $$

The intra-class correlation for individuals from the same genetic strain can be shown to be the proportion of the total variability explained by the genetic component:

3 $$  ICC = \frac{\sigma^{2}_{g}}{\sigma^{2}_{g}+\sigma^{2}_{\epsilon}}.  $$

However, such an interpretation is not straightforward for a non-linear model.

### Heritability for non-linear mixed models: variance partition coefficient (VPC)

Generalized linear mixed models (GLMM) are often used when the data are not Gaussian. For instance, a count data model such as the negative binomial with a log-link is a common choice for modeling RNA-Seq data. A GLMM version of Eq. 1 is as follows:

4 $${}  h(\mu)=\alpha + b_{s}, \; b_{s} \sim N\left(0,\sigma^{2}_{g}\right), \; E(y)=\mu, \; Var(y)=f(\mu).  $$

Here, the function *h* is the link function for the GLMM and the function *f* describes the mean variance relationship which depends on the assumed statistical distribution of the phenotype. Unfortunately, the additive property of the variance components does not hold for GLMMs.

Goldstein et al. [27] proposed a method to partition the variability in non-linear models. In a multi-level modeling setup, the authors describe a way to separate the variation due to the “higher level” (in our example, the strains) and any other sources of variability that remains. The Variance Partition Coefficient (VPC) is defined as the proportion of variability due to the “higher level” compared to the total variability. For instance, the total variation in *y* for the model given by Eq. 4 can be partitioned as

5 $$  Var(y)=Var(E(y|s)) + E(Var(y|s)).  $$

Here, the first term is similar to the between-strain-variance and the second term can be considered as average within-strain-variance. The VPC for this model is:

6 $$  VPC=\frac{Var(E(y|s))}{Var(E(y|s)) + E(Var(y|s))}.  $$

In the following sections, we propose four different methods for modeling the HTS data and derive the VPC in each case following the framework by Carrasco et al. [28] and Nakagawa and Schielzeth [29].

### Approaches for modeling HTS data

We will consider four different methods for estimating heritability from HTS data. Two of these methods are based on linear mixed models (LMM) after transformation of count data while the other two are based on GLMMs and therefore do not require the count data to be transformed.

Data obtained from HTS are counts ranging from 0 to 10,000s, and often contain many zero counts for features of interest (e.g., gene, miRNA) in particular samples. However, true zero inflated models were not considered because the example datasets examined here did not reflect explicit zero inflation (see Additional file 1: Section 1.1 and Figure S1).

We propose two GLMMs that can directly use the data without a transformation: (i) the compound Poisson mixed model (CPMM), which is a special case of the Tweedie distribution, and can model data using a continuous distribution with a mass at zero; (ii) the negative binomial mixed model (NBMM) which is the most popular choice for modeling HTS data due to its simplicity and ability to accommodate overdispersion. Both CPMM and NBMM allow for overdispersion, and we used a log-link in both cases. Below, we describe the LMM, NBMM and CPMM setups and derive the VPC in each case.

#### Linear mixed model (LMM)

Traditional LMMs can be applied to HTS data after the data have been properly transformed. A LMM assumes that the errors are normally distributed, and there are various ways to transform sequencing data so that the resulting data are approximately normal. We consider two popular transformations: (i) voom, in the limma **R** package [13] and (ii) a variance stabilizing transformation (VST), in the DESeq2 **R** package [30]. Both methods account for over-dispersion.

Suppose a dataset contains a total of *S* sample strains and *G* genes. Let *Y*_*gsr*_ be the observed number of sequencing read for the *g*th gene of sample *r* from strain *s*. *g*=1,⋯,*G*;*s*=1,⋯,*S*;*r*=1,⋯,*R*_*s*_, where *R*_*s*_ is the number of biological replicates within strain *s*. Denote the corresponding transformed read (either voom-transformed, LMM-voom, or VST-transformed, LMM-vst) as $Y_{gsr}^{*}$. The LMM can be expressed as the following:

7 $${} Y_{gsr}^{*} = \alpha_{g}^{*}+ b_{gs}^{*} + \epsilon_{gsr}^{*}, \;\; b_{gs}^{*}\sim N\left(0, \sigma^{2*}_{g}\right),\; \epsilon_{gsr}^{*}\sim N(0,\sigma_{\epsilon_{g}}^{2*}).  $$

For a fixed gene *g*, the intercept $ \alpha _{g}^{*}$ is shared among all samples, while the random effect $b_{gs}^{*}$ is strain specific. Furthermore, $b_{gs}^{*}$ and $\epsilon _{gsr}^{*}$ are assumed to be independently distributed. Under this model, the VPC for gene *g* is defined as

8 $${} {VPC}_{g}^{LMM} \!= \frac{Var(E(Y^{*}_{gsr}|s))}{Var(E(Y^{*}_{gsr}|s))+E(Var(Y^{*}_{gsr}|s))} \!= \frac{\sigma^{2*}_{g}}{\sigma^{2*}_{g} + \sigma^{2*}_{\epsilon_{g}}}.  $$

#### Negative binomial mixed model (NBMM)

Sun [14] showed that modeling the HTS data directly using count data models can be more powerful as compared to using linear models on transformed data. Also, the interpretation of VPCs as a measure of heritability becomes problematic if it is calculated from the transformed data. The following two aspects of HTS data were considered for deciding the choice of model.

It has been repeatedly shown that HTS data are overdispersed [31]. The negative binomial distribution has been the most popular choice to accommodate overdispersion. Methods such as edgeR [31], baySeq [32], and DESeq2 [33] use the negative binomial distribution in different ways to model HTS data. However, the existing methods using negative binomial model primarily concentrate on the analysis of differential expression rather than heritability and do not use mixed effects models. In other applications, ICC for NBMM has been used for reliability measurements [28, 34].

Under the NBMM, an observed number of reads aligned to gene *g*, *Y*_*gsr*_, follows a negative binomial distribution with mean *μ*_*gs*_ and variance $\mu _{gs}+\phi _{g} \mu _{gs}^{2}$, where *ϕ*_*g*_ is the dispersion parameter, shared across strains. The generalized linear model uses a log-link.

9 $$ \log(\mu_{gs}) = \alpha_{g}+ b_{gs}, \;\;b_{gs}\sim N\left(0, \sigma^{2}_{g}\right).  $$

Similar to the LMM, the intercept *α*_*g*_ is only gene specific and the random effect *b*_*gs*_ depends on both the genes and strains. The corresponding VPC for the *g*th gene is

10 $$\begin{array}{@{}rcl@{}} {}VPC_{g}^{NBMM} \!&=&\! \frac{Var(E(Y_{gsr}|s))}{Var(E(Y_{gsr}|s))+E(Var(Y_{gsr}|s))} \end{array} $$

11 $$\begin{array}{@{}rcl@{}} \!&=&\! \frac{Var(e^{\alpha_{g}+ b_{gs}})}{Var(e^{\alpha_{g}+ b_{gs}}) + E(\phi_{g} e^{2(\alpha_{g}+ b_{gs})}+e^{\alpha_{g}+ b_{gs}})} \end{array} $$

12 $$\begin{array}{@{}rcl@{}} \!&=&\! \frac{e^{\sigma^{2}_{g}} - 1}{e^{\sigma^{2}_{g}} - 1 + \phi_{g} e^{\sigma^{2}_{g}} + e^{-\alpha_{g}-\sigma^{2}_{g}/2} }. \end{array} $$

The last equality holds since $\phantom {\dot {i}\!}e^{\alpha _{g}+ b_{gs}}$ follows a log-normal distribution and the result is obtained using the mean and variance of that distribution. Note that $VPC_{g}^{NBMM} $ is strictly bounded above by $\frac {e^{\sigma ^{2}_{g}} - 1}{e^{\sigma ^{2}_{g}} - 1 + \phi _{g} }$, its range is therefore not [0,1], especially if gene *g* is overdispersed. Also it is almost always necessary to take into account different library sizes and possible batch effects for large studies, which results in post normalized data that are no longer integer counts. To use the NBMM the normalized data must be rounded off to the nearest integer.

#### Compound poisson mixed model (CPMM)

Some studies have shown that the negative binomial distribution might not always be the best model for HTS data [35, 36]. The compound Poisson distribution model provides an alternative and has been a popular tool in actuarial science and economy. However, few applications have been implemented in the field of biology and genetics. The compound Poisson model belongs to the family of Tweedie distributions that covers a relatively larger class of mean-variance relationships [37], and includes models like gamma regression or inverse Gaussian regression. It has the advantage of being able to model continuous post-normalized data while still accounting for potential zero inflation.

Under the CPMM an observed number of reads aligned to gene *g*, *Y*_*gsr*_, follows a compound Poisson distribution with mean *μ*_*gs*_ and variance $\phi _{g}\mu _{gs}^{p_{g}}$, for some 1<*p*_*g*_<2, where *p*_*g*_ is referred to as the Tweedie parameter. Under the CPMM, with a log-link, the regression on the mean has the same form as the NBMM:

13 $$ \log(\mu_{gs}) = \alpha_{g}+ b_{gs}, \;\;b_{gs}\sim N\left(0, \sigma^{2}_{g}\right).  $$

The VPC for gene *g* can be derived as

14 $$\begin{array}{@{}rcl@{}} VPC_{g}^{CPMM} &=&\frac{Var(E(Y_{gsr}|s))}{Var(E(Y_{gsr}|s))+E(Var(Y_{gsr}|s))} \end{array} $$

15 $$\begin{array}{@{}rcl@{}} &=& \frac{Var(e^{\alpha_{g}+ b_{gs}})}{Var(e^{\alpha_{g}+ b_{gs}})+ E(\phi_{g} e^{p_{g}(\alpha_{g}+ b_{gs})})} \end{array} $$

16 $$\begin{array}{@{}rcl@{}} &=& \frac{e^{\sigma^{2}_{g}} - 1}{e^{\sigma^{2}_{g}} - 1 + \phi_{g} e^{(p_{g}-2)\alpha_{g}+(p_{g}^{2}/2-1)\sigma^{2}_{g}}}. \end{array} $$

Similar to the derivation for $VPC_{g}^{NBMM}$, the final equality is a consequence of log-normal distribution properties.

### Testing the presence of heritability

The point estimate of the heritability score can be used to interpret the proportion of variability due to genetics. It is also useful to test whether genetics influence the variance of a specific trait (e.g., whether the expression of a particular gene is heritable). We propose a test for the null hypothesis that the heritability is 0 against the alternative that it is positive.

From the expression of the VPC for each model, clearly the VPC for gene *g* is 0 if and only if $\sigma ^{2}_{g}=0$. Conceptually, this is equivalent to the fact that the heritability will be zero if the variance component attributable to genotype is zero. Also, the VPC for each model is an increasing function of $\sigma ^{2}_{g}$ when all other model parameters remain constant. With indefinite increase of $\sigma ^{2}_{g}$, the VPC converges to its upper bound (1 for LMM and CPMM, $\frac {1}{1+\phi }$ for NBMM). We use a likelihood ratio test (LRT) to test

$$ H_{0}: \sigma^{2}_{g}=0 \;\; vs \;\; H_{1}: \sigma^{2}_{g}>0 $$

Since the null hypothesis lies on the boundary of the parameter space, the LRT statistic will follow a 50:50 mixture of $\chi ^{2}_{0}$ and $\chi ^{2}_{1}$ distributions [38], i.e. under *H*_0_,

$$ -2 \log(LR) \sim 0.5 \chi^{2}_{0} + 0.5 \chi^{2}_{1}. $$

The null can be accepted or rejected at specific levels by comparing the observed test statistic with the corresponding threshold of the mixture of chi-square distributions, and a *p*-value can also be computed. However, it should be noted that this method of hypothesis testing is not directly related with the point estimate of heritability using VPC. Therefore, the most significant features from the test may not be the same as the features with the highest heritability scores (more discussion in “Results” section).

### Confidence intervals for heritability

Finding confidence intervals for the heritability scores is challenging due to the complexity of the heritability score obtained using GLMMs and the inter-dependence among the model parameter estimates. Therefore, we have proposed a parametric bootstrap approach [39] to obtain the confidence intervals for both CPMM and NBMM approaches. For every fitted model, we bootstrap from the corresponding parametric family and fit the model. The appropriate percentiles of the bootstrap distribution are used as the lower and upper bounds of the confidence interval. We also proposed a third method using LMM-vst where we bootstrap from the model initially fitted using NBMM, but the bootstrapped data are fitted using LMM-vst. The justification of this approach lies in the fact that the variance stabilizing transformation assumes a negative binomial distribution. This method, although approximate in nature, has the advantage of being much faster than the CPMM and NBMM based bootstrap confidence intervals. We selected LMM-vst over LMM-voom due to the fact that the former appeared to be the superior method from each of our simulations (see Results).

### Implementation

Several existing **R**-packages have been used to implement the methods described. Packages glmmADMB (Version 0.8.3.3) [40] and lme4 (Version 1.1-12) [41] were used for fitting NBMMs and the package cplm (Version 0.7-4) [42] was used for fitting CPMMs. The LMM methods were fit using lme4 after transforming the data using the packages limma (Version 3.28.21) [43] or DESeq2 (Version 1.12.4) [33]. We have built an **R**-package HeritSeq that integrates all of these methods to estimate heritability, perform hypothesis testing, and provide confidence intervals. The package also includes functions to simulate data from each model.

### Data

The LXS RI panel reported by [19] originally consisted of 77 strains. With a well-controlled environment, the panel allows meaningful estimation of heritability of genetic traits. The miRNA and mRNA expression datasets used are based on a subset of the panel with multiple mice per strain. A miRNA is a small non-coding RNA containing about 22 nucleotides which promotes degradation or represses translation of target messenger RNA (mRNA). miRNAs are well conserved in both plants and animals, and are a vital and evolutionarily ancient component of gene regulation [44–48]. Estimating the heritability of the expression of each miRNA or mRNA can help reveal which are influenced by genetics.

miRNA data: Derived from [19], a total of 175 mice (57 LXS strains with 3 replicates and 2 strains with 2 replicates) were sacrificed and had total RNA extracted from whole brain tissues using the RNeasy Plus Universal Midi, Mini and miniElute kits for sequencing (Qiagen, Valencia, CA). The libraries were prepared using the Illumina TruSeq Small RNA Sample Prep kit. Fragments between 20–35 bp were selected and the libraries were sequenced on the Illumina HiSeq 2500 platform. The size selected small RNAs were then mapped using a novel k-mer matching method to quantify the number of sequencing reads per individual miRNA. Following mapping and quantitation, normalization and batch correction were performed (see Additional file 1: Section 1.2 and Figures S2–S4).

mRNA data: The original mRNA dataset includes a total of 236 samples (42 LXS strains) with either saline or ethanol treatment [49]. We worked with the saline treated samples that have at least two biological replicates per strain. The resulting mRNA dataset contains 118 samples (40 LXS strains, 2 to 3 replicates each). Total RNA was extracted from whole brain tissue using RNeasy Mini Kits (Qiagen, Valencia, CA), quantity and quality were determined using a NanoDrop spectrophotometer (Thermo Fisher Scientific, Wilmington, DE) and Agilent 2100 BioAnalyzer (Agilent Technologies, Santa Clara, CA). The libraries were prepared using Illumina ScriptSeq RNA-Seq Library Preparation Kit v2. Sequencing was performed on the Illumina HiSeq 2000 platform. Details on generation of strain-specific genomes can be found in the Additional file 1: Section 1.3. Each LXS sample was aligned with Tophat2 (v2.0.6) to its strain-specific genome. Gene quantification was performed using HTseq (v0.6.1) to obtain RNA fragment counts over each annotated gene. The dataset was normalized without any batch correction, since there was no noticeable batch effect.

Both miRNA and mRNA features were filtered to eliminate those with small counts on most samples. For miRNA we required at least 5 samples to have at least 10 counts; for mRNA we required at least 2 samples to have at least 10 counts. We chose 5 to be the number of minimum samples because the data from parental strains were also obtained, and each parental strain had at least 5 samples. As for the mRNA data, only LXS samples were available to us; each strain had at least 2 samples. After filtering, the LXS miRNA and mRNA datasets have 881 and 17537 features, respectively. We then adjusted read counts for effective library size using the **R**-package DESeq2.

### Simulations

To compare the performance of the four methods: NBMM, CPMM, LMM-vst, LMM-voom, we created five types of simulations (Table 1). The first type (*Simulation I*) explores the behavior of each approach for every combination of model parameters. The goal is to investigate if certain combinations of parameters result in more precise estimates of heritability. The second type (*Simulation II*) generates a more realistic full dataset where each feature is based on a distinct combination of model parameters. This simulation checks how the heritability methods compare across many features that are based on independent and unrestricted combinations of parameters. The third type (*Simulation III*) is based on the model parameter combinations observed when modeling the preprocessed LXS RI miRNA dataset. The simulated datasets in this case reflect the properties of the LXS RI miRNA dataset, including potential dependence among the features. The last two simulations compare the four methods through hypothesis testing for the presence of heritability. The fourth type (*Simulation IV*) used to investigate the type-I error and power and the fifth type (*Simulation V*) compares the confidence intervals using bootstrap. Table 1 provides an overview of the simulation setups. For each simulation, we generated datasets either under NBMM or CPMM to examine the performance under model misspecification. To distinguish the data generating model and the fitting model, for the rest of the paper we will use NB-sim and CP-sim explicitly for data generation; NB-fit and CP-fit will be used to denote fitting methods being compared. The methods LMM-vst and LMM-voom will be simply referred to as VST and voom.

**Table 1:** Simulation setup summary

Simulation	(*ϕ*,*α*) *or* (*p*,*ϕ*,*α*)	*σ* ^2^	(*G,S,R*_*s*_)
I. Parameter effects	Constant for all features	Random samples from Unif$(0, \sigma ^{2}_{\max })$, where $\sigma ^{2}_{\max }$ = 1 or 5	(1000, 50, 6)
II. Exhaustive combo of parameters	Ind. combo of parameters for every feature	Random samples from Unif (0,5)	(1000, 50, 3)
III. Observed combo of parameters	Estimated from the LXS miRNA dataset		(881, 59, 2 or 3)
IV. Size & power	Estimated from the LXS miRNA dataset	0,0.1,0.25,0.5,0.75 or 1	(1000, 50, 3)
V. Confidence intervals	Specifically chosen to generate heritability scores 0.2, 0.5 and 0.8		(500, 50, 3)

The parameters (*ϕ*,*α*) and (*p*,*ϕ*,*α*) are for NB-sim and CP-sim, respectively; *σ* ^2^ denotes the random effect variance in either model. The last column shows the number of features (*G*), strains (*S*), and biological replicates (*R*_*S*_) in each simulation. Under each scenario, we simulated data using both NB-sim and CP-sim models. Simulation II and III include 10 replicated synthetic datasets for each case

#### Simulation I: evaluate influence of different parameter combinations

We generated datasets from different sets of model parameters to see how the four methods perform under different situations. The true distribution was assumed to be either negative binomial or compound Poisson. For each scenario, we fixed the parameters for negative binomial or compound Poisson distribution, i.e. we had the same *α*_*g*_ and *ϕ*_*g*_ (or the same *α*_*g*_, *p*_*g*_ and *ϕ*_*g*_) for each *g*, but varied $\sigma ^{2}_{g}$ to generate different heritability scores for different features. For each scenario, we simulated 1000 features. For each feature, we simulated data for 300 samples (50 strains and 6 samples per strain). We computed the true VPC for each feature and compared these to the estimated VPCs from the proposed methods. While using the VST or voom transformations, we treated the 1000 features as one dataset. The parameters specific to the NB or CP distributions were chosen from the range of the estimated parameters when the models are fitted to the LXS-miRNA data. The $\sigma ^{2}_{g}$ values were simulated from a $U(0,\sigma ^{2}_{max})$ distribution where $\sigma ^{2}_{max}$ was chosen to be either 1 or 5.

#### Simulation II:

In a second set of simulations we sought to examine the behavior of the proposed methods in a more realistic setting where an entire dataset of features with a wide variety of parameter combinations were simultaneously analyzed. One of the main reasons this simulation paradigm was investigated was the fact that both of the transform methods (VST and voom) rely on the entire dataset in their underlying algorithms, so we sought to give them an opportunity that would better replicate the scenario they were designed for. This was done under both the NB-sim and CP-sim models where each feature had a random set of parameters drawn from an appropriate distribution (see Additional file 1: Section 1.4 for details).

The dataset generating functions also allow for some proportion of features to be simulated from a null model that has a heritability that is identically equal to 0 (i.e. the random effect variance $\sigma ^{2}_{g} = 0$ and thus all random effects are identically 0). A final option was the specification of a proportion of features that have a high heritability. This is achieved in general by forcing a small dispersion value *ϕ*_*g*_ and a larger random effect variance $\sigma ^{2}_{g}$.

Using this algorithm, we generated 10 datasets each under 4 combinations of the data generating model and number of strains S. In all datasets the number of features *G* was set at 1,000 and the number of replicates *R*_*s*_ was fixed at 3. For both NB-sim and CP-sim data we examined two values for the number of strains *S*, 25 and 50. In each of the 40 simulated datasets, 10% of the features were simulated with 0 heritability and 10% of the features were simulated with high heritability. The remaining 80% were simulated with independent draws of the model parameters (Additional file 1: Section 1.4). All four of the proposed methods for assessing heritability were fit on each dataset.

#### Simulation III: LXS miRNA dataset based

Datasets generated in Simulation III mimic the LXS miRNA data described in “Data” section. The model parameters are obtained from fitting GLMMs to the preprocessed LXS miRNA dataset. Details on preprocessing this dataset are discussed in Additional file 1: Section 1.2. We simulated a new dataset based on the estimated parameters from the regressions under NB-sim and CP-sim. Both retain the same mean-variance relationships as the processed dataset Additional file 1: Figure S4. Simulating based on real data also retains the dependencies among parameters. Each generated data matrix has the same number of samples per strain (*R*_*s*_,∀*s*=1,⋯,*S*.) and the same number of genes (*G*) as the original LXS miRNA dataset. The simulation procedure was repeated 10 times with different random seeds.

#### Simulation IV: power and type-I error of the hypothesis test

We performed a set of simulations to compare the power and type-I error of the four methods for hypothesis testing. The true distribution was assumed to be either NB or CP. Datasets with 1000 features, 50 strains and 3 samples per strain were simulated. For each feature, the parameters of the NB (or CP) distribution were selected at random with replacement from the sets of parameters estimated from the LXS data (from the first step of Simulation III). A fixed value of $\sigma ^{2}_{g}$ was used for each dataset. We considered six such fixed values: 0 (null hypothesis), 0.1,0.25,0.5,0.75, and 1. The goal was to investigate how the power of the tests increase as the strain specific variance component increases. The tests were performed at 5% level, and the type-I error and power of the tests were recorded.

#### Simulation V: confidence intervals

In this final set of simulations, we investigated the coverage of our proposed interval estimation methods. The goal of this simulation was to study the coverage of the confidence intervals for low (0.2), medium (0.5) and high (0.8) heritability features. Using NB-sim, we simulated 500 features for each of the three levels of heritability and computed the proportion of cases where the true heritability is covered by the estimated confidence interval using the NB-fit, CP-fit, and VST methods. The analysis is repeated using data generated from the CP-sim model.

## Results

In this section we compare the VPC computed via the four methods: NB, CP, VST, and voom. The performance of each method in the simulations is evaluated by comparison to the true VPC values. For the real data implementation, we show the pairwise comparisons between the top methods, as well as the estimated score distributions.

### Simulation I: evaluate influence of different parameter combinations

**Fig. 1:**
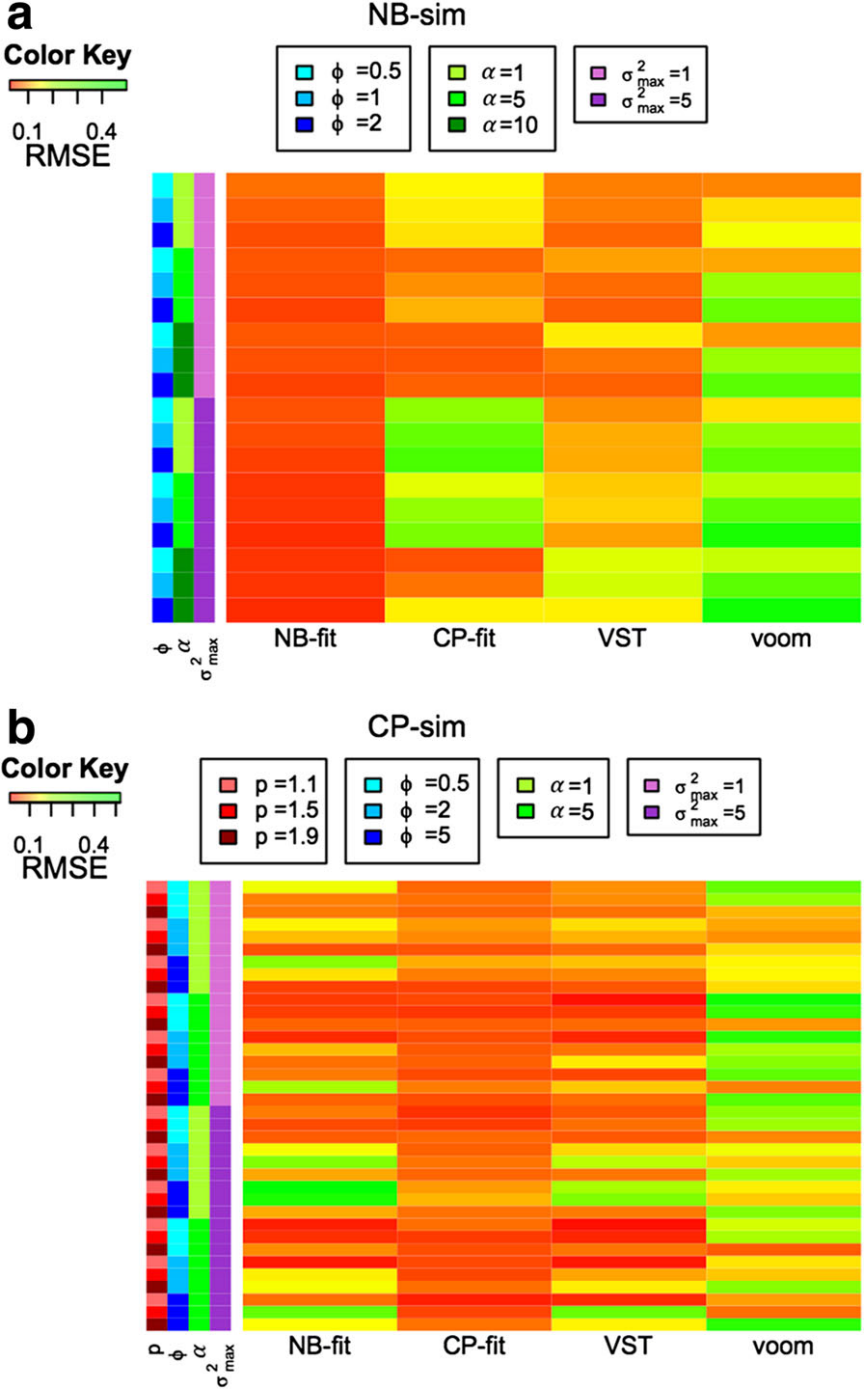
Comparison of heritability precision for different combinations of true model parameters. (Simulation I). Heatmap of Root Mean Square Error (RMSE) corresponding to the four methods for estimating VPC. The data are generated from **a** NB-sim **b** CP-sim. The parameter combinations for the simulation are shown by the sidebars, the *darker* the color in the sidebar, the higher the value of the parameter. In the heat map, *red* indicates better performance (small RMSE) compared to *green*

From the simulation results, we observed that the performance of the method is largely driven by the amount of overdispersion *ϕ* and the strength of strain-specific variance $\sigma ^{2}_{g}$ (Fig. 1). When data were generated from the same model that estimates the heritability, precision is maximized. The range of the RMSE is [0.03,0.07] for NB-fit and [0.02,0.11] for CP-fit in such cases. Under model misspecification (CP-sim generated data), the performance of NB-fit suffers when the amount of over-dispersion (*ϕ*) is large and *p* is small. While with NB-sim generated data, CP-fit usually suffers when the strain specific variance $\sigma ^{2}_{g}$ is large. The VST approach appears to be quite robust against the choice of the true distribution, but it is usually less accurate than the model that is used to generate the data. VST has the second best performance in 72% of the cases under NB-sim and in 58% of the cases under CP-sim. It has low RMSE for NB-sim data (maximum RMSE =0.17) and is highly correlated with NB-fit for CP-sim data (correlation coefficient =0.86). The performance of voom is not satisfactory (mean RMSE under NB-fit and CP-fit are 0.25 and 0.20 respectively), especially when the data comes from a highly over-dispersed negative binomial distribution. Additional file 1: Figures S5 and S6 show the comparison of different methods for the two true distributions under high and low dispersion situations. It is evident that when the true distribution is CP, NB-fit may under-estimate the heritability. On the other hand, when the true distribution is NB, CP-fit may result in over-estimation of the heritability.

We used a relatively larger number of samples per strain (6) for this simulation set up since we wanted to investigate how the methods perform in a relatively ideal situation. However, we also carried out the simulations for 3 samples per strain and the results were similar (data not shown).

### Simulation II: more realistic sequencing data

**Fig. 2:**
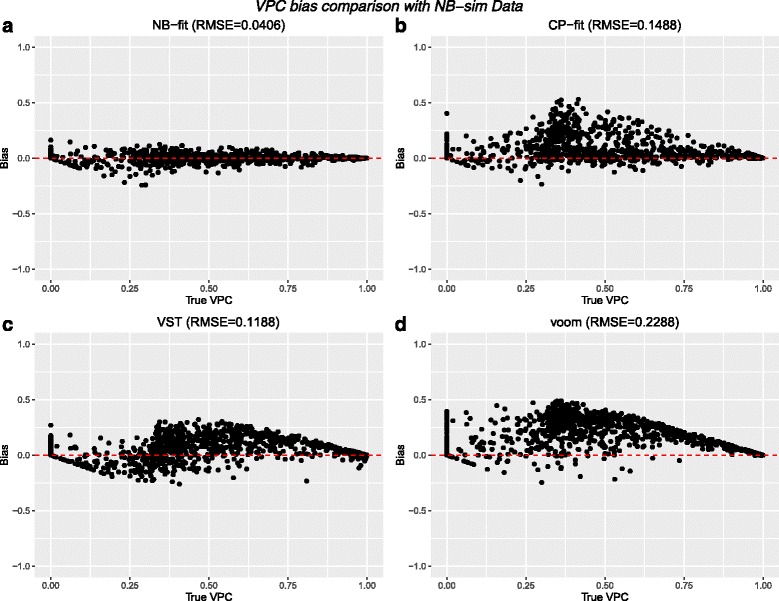
Bias comparisons for the four methods for a single NB-sim dataset with 1000 features, 50 strains, and 3 replicates per strain. Bias is measured as estimated VPC − true VPC

When looking at the results based on the entire simulated datasets, we again see the pattern of NB-fit, CP-fit, and VST methods generally agreeing with each other and the true values, while the voom transform struggles to recover the true heritability regardless of whether the data were generated using NB-sim or CP-sim (Fig. 2). We found that the NB-fit estimates clearly perform best with both the smallest average bias across all 10 simulated datasets (0.0005) and RMSE. The next best performing method was the VST with an average bias of 0.0560, followed by the CP-fit with an average bias of 0.0876. As in Simulation I, the voom transform performed the worst of the methods examined here with an average bias of 0.1822. When looking for patterns in the bias for each method we see no systematic trend when using the NB-fit with NB-sim data, but the CP-fit appears to almost always overestimate the VPC on such data. The VST shows underestimation for smaller values of the true VPC while then switching to overestimating in the larger values. The voom transform also tends to strictly overestimate the VPC throughout the range of true values, and also tends to give larger values to the features that were simulated with a VPC of 0 (stacked dots above 0 in Fig. 2). In terms of classifying features as heritable or not, the AUC from creating ROC curves based on VPC threshold of 0.5 was strong for all 4 methods. The best performer was again the NB-fit with an AUC of 0.996 while the CP-fit had the lowest AUC of 0.935. This general behavior for all the methods was repeated in each of the 10 simulations using the NB-sim data with *S*=50, as well as when *S*=25 (data not shown). Adding in the additional strains in the 10 simulations with *S*=50 did not qualitatively alter the performance of the methods in terms of average bias or RMSE from what was observed in the simulations with *S*=25.

When using CP-sim data with *S*=50, we saw that the ordering of the methods in terms of average bias and RMSE changed similarly to what was observed in Simulation I in that now CP-fit performs the best in terms for average bias and RMSE, followed by VST, NB-fit, and then voom (Additional file 1: Figure S7). The same pattern held for AUC analysis using a 0.5 heritability threshold. The only major difference from the NB-sim results is that now all of the methods tended to underestimate the VPC on CP-sim data, and no method could match the average bias or RMSE from the NB-fit on NB-sim data. As was observed with the NB-sim datasets, the average bias, RMSE, and AUC estimates for all methods were virtually identical when both *S*=25 was used (data not shown).

### Simulation III: LXS dataset based

**Fig. 3:**
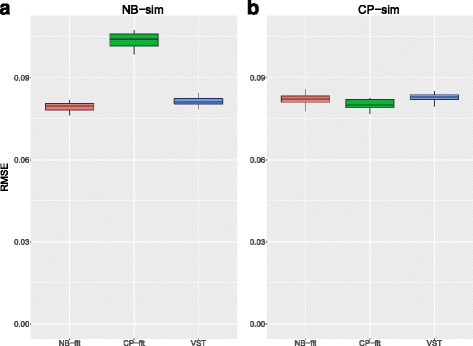
Accuracy comparisons for Simulation III. RMSE for NB-fit (salmon), CP-fit (*green*), and VST (*blue*) across 10 simulated dataset generated based on either **a** NB-sim or **b** CP-sim. The true positives are defined as miRNAs with true VPC greater than 0.5

Although the different preprocessing procedures (Additional file 1: Section 1.2) have limited effect on the VPC estimation, the results do depend on which GLMM was used to generate the dataset. Similar to previous simulations, NB-fit, CP-fit and VST show good performance while the voom approach is worst at estimating heritability. Therefore, we omit the results from voom and focus only on NB-fit, CP-fit and VST from now on. The method that matches the true underlying model is best at distinguishing heritable features as defined by having a VPC>0.5 (AUC = 0.99 for the NB-fit on the NB-sim data; AUC = 0.99 for the CP-fit on the CP-sim data, also see Fig. 3). Under model mis-specification, NB-fit (AUC = 0.98; median RMSE = 0.0822) appears to be more robust than CP-fit (AUC = 0.97; median RMSE = 0.1041), while VST shows fairly robust result for both sets of simulated data (AUC = 0.99 for NB-sim; AUC = 0.98 for CP-sim).

### Simulation IV: power and type-I error of the hypothesis test

When the data were simulated from CP-sim, power for the CP-fit and NB-fit methods were very close (Table 2). For the NB-sim data, CP-fit method had higher power, but it also had inflated type-I error (estimated type-I error = 0.10). The CP-fit method can over-estimate heritability when the data are generated from NB-sim. The VST method appeared to be conservative and hence less powerful in both cases. The voom method had low power for detecting smaller departures from null, but was quite good for large departures (data not shown).

**Table 2:** Type-I error and power (*α*=0.05) of the four methods for data simulated from NBMM and CPMM

	Data from NB-sim						Data from CP-sim					
Method/ $\sigma ^{2}_{g}$	0	0.10	0.25	0.50	0.75	1	0	0.10	0.25	0.50	0.75	1
CP-fit	0.10	0.78	0.89	0.94	0.96	0.97	0.04	0.72	0.86	0.92	0.96	0.96
NB-fit	0.04	0.71	0.86	0.92	0.95	0.95	0.04	0.72	0.86	0.92	0.96	0.96
VST	0	0.56	0.76	0.86	0.90	0.92	0	0.55	0.76	0.86	0.90	0.92

The value in a cell denotes the value of the power function (rounded off to 2 places after decimal) for each case. The value of $\sigma ^{2}_{g}$ drives the power. The case $\sigma ^{2}_{g}=0$ reflects the null hypothesis, therefore corresponding the cell value is the type-I error. Columns with $\sigma ^{2}_{g} > 0$ indicates the power for increasing strain specific variance

### Simulation V: confidence interval

The results of the confidence interval simulation (Table 3) indicate that the confidence interval coverages of all the methods are slightly below the target 95% for NB-sim data. The CP-fit based method has higher coverage compared to NB-sim for all true heritability levels. For CP-sim data, the coverage of all three methods are similar for low heritability, the percentage coverage being slightly smaller than 95%. However, the coverage percentage becomes lower for NB-fit and VST based methods as the true heritability score increases. The coverage of CP-fit stays at the same level. The coverages of NB-fit and VST are significantly less than 95% for high heritability when data are generated from CP-sim. The coverage of VST is the highest for low heritability, but not satisfactory for higher heritability under CP-sim.

**Table 3:** Coverage of the VPC by 95% confidence intervals (based on 500 simulations)

	Data from NB-sim			Data from CP-sim		
Method/ True VPC	Low	Medium	High	Low	Medium	High
CP-fit	91.8 (0.32)	93.2 (0.32)	94.2 (0.19)	93.4 (0.32)	92.4 (0.33)	92.8 (0.20)
NB-fit	90.2 (0.31)	91.8 (0.32)	92.0 (0.18)	93.4 (0.31)	90.4 (0.31)	78.4 (0.19)
VST	92.0 (0.32)	91.6 (0.33)	92.4 (0.20)	94.8 (0.32)	89.6 (0.34)	84.0 (0.22)

Coverage percentage is shown at each level of true heritability score for data generated from NB-sim and CP-sim. Trues VPCs 0.2, 0.5 and 0.8 were considered as representatives for low, medium and high heritability. The values in parentheses show the average lengths of the intervals for the different cases. 200 bootstraps are used in each case

The average lengths of the confidence intervals are very similar across methods, approximately 0.3 for low and medium heritability, and approximately 0.2 for high heritability. We have also observed that in most the cases where the confidence interval does not cover the true heritability, the interval underestimates (i.e. the upper limit of the interval is smaller than the true value). The proportions of underestimation among all non-coverages for NB-fit based method are 75% (NB-sim data) and 97% (CP-sim data). The same percentages are 80% and 97% for VST, and 69% and 78% for CP-fit (See Figure S8 for a representative example).

### Application to the LXS RI miRNA dataset

**Fig. 4:**
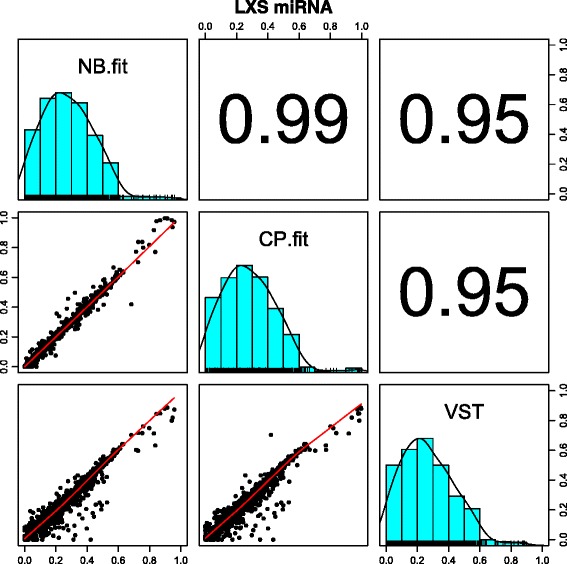
Heritability comparison across estimation methods for the LXS miRNA preprocessed dataset. The histograms with rug (tick marks along the x-axis) and kernel density plots are shown along the diagonal. The *panels below* the diagonal show the scatter plots and the LOESS fits for the pairwise comparisons. The corresponding correlation values are listed *above the diagonal*

For the real data application, we observed similar result as in the simulations. The NB-fit, CP-fit, and VST methods produce highly correlated heritability estimates (Fig. 4). The locally weighted scatterplot smoothing (LOESS) fits (red lines in Fig. 4) are roughly linear. The overall VPC distributions are very similar among the methods. The result for voom is omitted here due to poor performance. Since VST is a negative binomial based transformation, it is not surprising that the results from NB-fit and VST are highly consistent (*ρ* = 0.95), as was observed in the simulations. The CP-fit estimators are also highly correlated with NB-fit results (*ρ* = 0.99). The overall distribution of heritability estimates have similar shape for NB-fit, CP-fit, and VST; they are all right-skewed. However, the range of estimates differs slightly between methods due to theoretical ranges (see Section “Methods” section).

We also tested the presence of heritability using each method and looked at the distribution of the corresponding p-values (not shown here). The *p*-value distributions are more similar between the two GLMM (NB-fit & CP-fit) and between the two LMM (VST & voom). This is a consequence of modeling based on the original data versus the transformed data and is consistent with the power comparison results. At False Discovery Rate (FDR) level 0.001, the four methods NB-fit, CP-fit, VST, and voom respectively report 471 (53%), 475 (54%), 306 (35%), 304 (35%) miRNA features with evidence of heritability ($\sigma ^{2}_{g} \neq 0$). Although the heritability estimation and the hypothesis testing use two different statistics and are not expected to have matching results, the rank correlations between *p*-value and heritability estimate within a method are very high except for voom, which indicates that for the other three methods the score estimation and testing substantially agree with each other (Additional file 1: Section 2.1 and Figure S9).

In addition, we conducted the analysis for CPMM preprocessed data and the comparison of heritability estimates across methods are similar (Additional file 1: Figure S10). Regardless of the preprocessing methods, the top heritable features identified coincide (Table 4). Some of the miRNAs present a continuous spectrum of reads while others exhibit bimodal read distributions (Additional file 1: Figure S11). The feature mmu-miR-446q has a high heritability score without the bimodal trend (Fig. 6 a). Such sequencing read distribution were also observed for several other features, suggesting that those corresponding miRNAs are likely to have an eQTL.

**Table 4:** Top heritable miRNA based on the LXS dataset

miRNA	VPC (NB)	*p*-value	VPC (CP)	*p*-value
novel:chr10_26214	0.959	2.2e–39	0.978	9.6e–38
mmu-miR-5621-5p	0.947	1.2e–27	0.955	1.1e–28
mmu-miR-466q	0.941	1.4e–22	0.982	1.5e–21
mmu-miR-9769-3p	0.914	8.5e–33	0.994	1.6e–33
novel:chr4_11381	0.898	2.6e–31	0.996	8.8e–28
novel:chr8_23508	0.867	1.8e–25	0.994	1.8e–27
mmu-miR-7057-5p	0.844	5.5e–27	0.979	5.3e–25

The second and fourth columns are the VPC scores for the datasets processed and fit under NBMM (NB-proc & NB-fit) and processed and fit under CPMM (CP-proc & CP-fit), respectively. The corresponding p-values for testing the presence of heritability are listed in the adjacent columns. The features are sorted by their heritability estimates using the NB-fit method

From a separate eQTL study on the same data using CPMM model (Additional file 1: Section 1.5), we found that all of the top 7 heritable miRNAs had an eQTL when tested at FDR =0.05. While there were several moderately heritable miRNA features which did not have an eQTL, the results show that the highest heritability estimates were observed in cases where a single nucleotide polymorphism (SNP) is strongly associated with the miRNA expression. However, there were several miRNAs with high heritability score, but no eQTL at the above threshold. This shows the utility of the heritability analysis to find features with high genetic variation that eQTL analysis alone cannot detect, one possible reason being the weak association of miRNA expression with multiple SNPs.

### Application to the LXS RI mRNA dataset

**Fig. 5:**
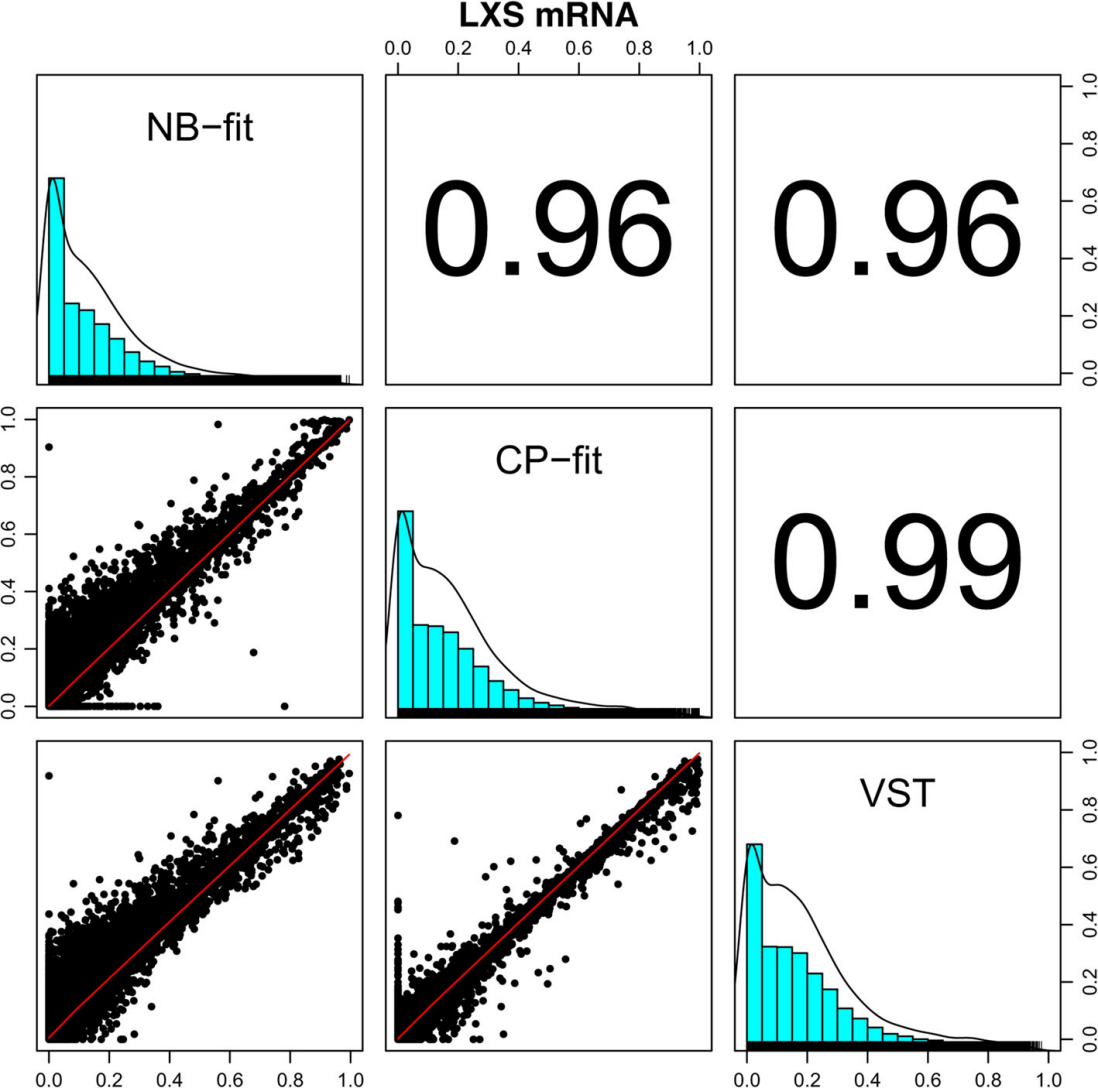
Heritability comparison for the LXS mRNA dataset. The histograms and kernel density plots are shown along the diagonal. The *panels below the diagonal* show the scatter plots and the LOESS fits for the pairwise comparisons. The corresponding correlation values are listed *above the diagonal*

As with the miRNA data, NB-fit, CP-fit, and VST show highly consistent results for the LXS mRNA data (Fig. 5). There are a few features that exhibit zero VPC scores under the CP method while displaying small heritability under other models. This is an artifact caused by the optimization algorithm for regression fitting. It does not affect the identification of moderately or highly heritable features. Similar to the miRNA dataset, the overall distribution of estimated heritability scores have nearly identical shape for NB-fit, CP-fit, and VST. They are all right-skewed and peak near 0. The distributions are much more narrow compared to the miRNA data. The range of the estimated heritabilities differs between methods, but not as much as with the miRNA. This could be due to the fact that the mRNA dataset includes 16851 features while the miRNA data only have 881 features. At FDR level 0.001, the number of features with significant presence of heritability are 2470 (15%), 2633 (16%), 1763 (10%), and 1850 (11%) for methods NB-fit, CP-fit, VST, voom, respectively.

**Fig. 6:**
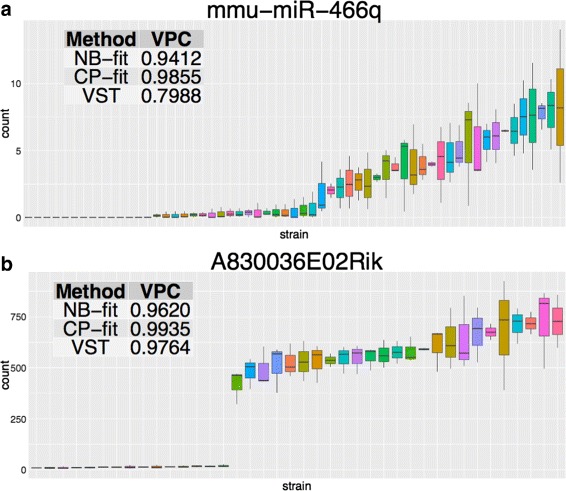
Sequencing read examples for **a** a top heritable miRNA and **b** a top heritable mRNA features. Each boxplot summarizes the reads for one strain and they are sorted by strain mean in an increasing order. The color of the boxes have no special significance. The estimated VPC scores are reported in the *top left* tables

Based on NB-fit, heritability estimate of 16 genes exceeds 0.95 threshold (Additional file 1: Section 2.2 and Table S1). Two of them show continuous read distribution trend, while the others are bimodal (figure not shown here). We also examined the top 30 heritable features using either NB-fit, CP-fit, or VST models and found 7 genes that appear across the list: A830036E02Rik, Ccl28, D030028A08Rik, Exoc3, Gm5148, Gm21967, Krt12. Like gene A830036E02Rik (Fig. 6 b), all of them have bimodal sequencing read distributions. The read distributions, their corresponding heritability estimates and p-values are reported in the Additional file 1: Table S2 and Figure S12.

## Discussion

Heritability is an important concept in evolutionary biology and relevant for breeding in agriculture and animal studies. It can be useful to gain insight on the genetic basis of individual traits [50] and is therefore a critical concept in the prediction of disease risk in medicine [4]. When the traits of interest are gene expression, a related analysis is the detection of eQTLs. The top heritable results we report have a bimodal pattern, which is likely a result of a single strong eQTL. Despite this overlap, heritability and eQTL analysis also give complementary information. We found that other highly heritable miRNA features did not show a bimodal pattern, and may be a result of associations from multiple loci more difficult to detect in eQTL analysis [51, 52].

While we did observe this bimodal distribution in many of the features with the highest heritability measures, we do not believe that it necessitates the use of a zero-inflated model. For a given miRNA or mRNA that showed this bimodal distribution of counts, virtually all of the small or zero counts were observed in samples from strains that had means close to zero. The zero counts were not equally distributed across all of the strains, and thus we found that the strain-specific means estimated using the random effects within the GLMMs (i.e. NBMM and CPMM) were able to capture the observed behavior.

A crucial element for proper heritability estimation is the use of a genetically well characterized population. We take advantage of RI panels maintained in controlled environments. The generalized linear models discussed here may be used for other types of experiments that are not based on RI panels, but with modifications. Even though mixed effects models may be appropriate for repeatedly measured data such as longitudinal data, the variance partition coefficient may not always be suitable as a measure of heritability. The user should be careful about the difference between heritability and repeatability where the latter measures the relatedness of the repeated observations [26, 53]. However, for a recombinant inbred panel, the only common factor among the strains are their genetic background. Animals from the same strain will have a shared environment in a well designed animal study. Thus, the proportion of strain specific variation can accurately measure the heritability. If our methods are to be used for other types of populations, one should make sure that there is no other common factor other than the genetic factor among the subjects within a class.

Among the four methods proposed in this work, the NB-fit, CP-fit and VST methods perform similarly and all of them have very high accuracy. However, voom failed to perform well in many cases and appears to be useful only in limited situations. voom’s performance is especially limited when the data are overdispersed and not highly heritable. CP-fit and NB-fit are most accurate when the data are generated from the respective distributions. The estimation performance of NB-fit is slightly better than CP-fit under model-misspecification. The estimation using VST is the most robust against model-misspecification and it performs the second best in most cases.

The hypothesis testing procedure using CP-fit may have anti-conservative results when the data are NB-sim. The test using NB-fit is slightly conservative, but sufficiently powerful under model-misspecification. Tests using VST are the most conservative and hence the least powerful. The CP-fit based bootstrap confidence interval performs the best among the three different methods considered. All the confidence intervals have a tendency to underestimate the true heritability, but the CP-fit based method seem to be the least biased and have coverage closest to the target 95%. These bootstrap based confidence intervals are computationally expensive and one may choose to use them only for a limited number of interesting features. In terms of computational cost (both estimation and hypothesis test), CP-fit is faster than NB-fit, and the LMM methods (VST and voom) are much faster than both CP-fit and NB-fit. For the LXS miRNA dataset with hypothesis testing, the CPU time required was 17.167, 12.607, 0.265, 0.301 for NB-fit, CP-fit, VST, and voom, respectively (OS X 10.10.5, 2.5 GHz Intel Core i7, 16 GB 1600 MHz DDR3).

Each method has additional considerations as well. One limitation of the NB-fit based heritability score is that it needs to be interpreted with caution because the maximum possible heritability score is less than 1 for over-dispersed data. This is a direct consequence of the algebraic expression of $VPC_{g}^{NBMM}$. For the same reason, the heritability score for a feature may have a smaller value when using NB-fit as compared to CP-fit. Although CP-fit does not show any significant increase in accuracy to estimate the heritability for data similar to our count data, it might be appropriate for other types of post-normalized data with many zeros or heavy tails (e.g. for sparse data like microbiome). We showed VST is robust and in many cases sufficiently accurate while voom results can be misleading. Both methods fit the model on a completely different scale due to data transformation which makes the interpretation of the heritability score more problematic than the NB and CP methods.

In summary, we suggest to use VST, NB-fit, CP-fit, and compare the results. The three methods have different strengths. Computationally, VST is the most efficient and robust; the NB-fit method performs well in hypothesis testing even under model mis-specification; the CP-fit based confidence intervals are the most reliable. The choice of one method out of the three should depend on the goal of analysis.

## Conclusions

In this work, we have proposed several statistical models and methods for estimating and testing heritability for high-throughput sequencing data and have provided an R package HeritSeq implementing our methods. Although mixed effects models have been used in the context of repeatability [29], we studied the rather unexplored area of calculating heritability for non-Gaussian or count based data. Our work reports the use of the variance partition coefficient to extend the definition of heritability for generalized linear mixed models in the context of sequencing data. The variance partition coefficient is conceptually different from traditional measures such as intraclass coefficient and is more suitable for measuring heritability in this context. We have proposed the use of the CP mixed model which has not been previously used for genomic data. Through simulations and two sets of sequencing data from an RI panel, we demonstrate that NB-fit, CP-fit, and VST are better methods for estimating heritability than the voom method. For a miRNA and mRNA expression dataset, we identified heritable features and found that many of the highly heritable features exhibit bi-modal sequencing counts, which are likely expression quantitative trait loci. In summary, the ability to better model high throughput sequencing data and estimate the heritability scores will elucidate the functional mechanisms in genetic networks.

## Additional file

Additional file 1 Supplementary materials. (PDF 1370 KB)

## Abbreviations

ANOVA: analysis of variance
CP-fit: compound Poisson mixed model fit
CP-sim: expression data simulated under compound Poisson mixed models
CPMM: compound Poisson mixed model
eQTL: expression of quantitative trait loci
HTS: high-throughput sequencing
ICC: intra-class correlation
ILS: inbred long sleep
ISS: inbred short sleep
GLMM: generalized linear mixed model
LMM: linear mixed model
LXS: recombinant inbred of long-sleep and short-sleep mice
miRNA: microRNA
mRNA: messenger RNA
NB-fit: negative binomial mixed model fit
NB-sim: expression data simulated under negative binomial mixed models
NBMM: negative binomial mixed model
RI: recombinant inbred
RNA-Seq: high-throughput sequencing studies of RNA
VPC: variance partition coefficient
VST: variance stabilizing transformation
