# Supplementary material for: Model based heritability scores for high-throughput sequencing data
Source: BMC Bioinformatics. 2017 Mar 2;18:143. doi: 10.1186/s12859-017-1539-6 (PMC5333443; doi:10.1186/s12859-017-1539-6)
Supplement: Supplementary file 1 — Supplementary materials. (PDF 1370 KB) [file 12859_2017_1539_MOESM1_ESM.pdf]

METHODOLOGY

Model based heritability scores for high-throughput sequencing data: Supplementary Material

Pratyaydipta Rudra[1†], W. Jenny Shi[2†], Brian Vestal[1†], Pamela H. Russell[1], Aaron Odell[3], Robin D. Dowell[4,5], Richard A. Radcliffe[6], Laura M. Saba[6] and Katerina Kechris[1*]

*Correspondence:
katerina.kechris@ucdenver.edu
[1]Department of Biostatistics and Informatics, University of Colorado School of Public Health, CO 80045 Aurora, USA
Full list of author information is available at the end of the article
†Equal contributor

1 Supplementary Methods

1.1 Zero inflation

The LXS-panel miRNA expression data had many zero counts even after the normalization and filtering steps. We considered fitting zero-inflated models such as zero-inflated negative binomial mixed model (NBMM) was made. However, fitting a zero-inflated NBMM is computationally intensive and none of the miRNAs in our data showed the typical behavior of a zero inflated negative binomial distribution with two modes (Figure S1). The zeros appear to be generated from a distribution with low mean rather than being generated from a different source. Therefore, we decided against the use of zero-inflated negative binomial model.

1.2 LXS miRNA mapping and quantitation

Naive mice (no alcohol exposure) were sacrificed using CO_2 gas at approximately 10 weeks of age and brains were removed, divided sagittally, and placed in RNALater (Applied Biosystems/Ambion) for RNA extraction and quantitation.

Due to the challenges of mapping miRNA-derived reads to the genome, where many mature miRNAs are present in multiple copies, genome-based quantification methods are often inappropriate for miRNAs. Furthermore, transcriptome-based methods often feature implementation details that implicitly assume that transcripts are much longer than the sequencing reads, causing unexpected behavior when these methods are used for small RNAs. For these reasons, we have developed a novel quantification method for miRNAs. We searched for perfect 20nt matches between reads and mature miRNA sequences. This level of stringency was made possible by approximating individualized genomes for each sample: we combined DNA sequencing in the two parental strains with SNP genotyping in all RI strains to call dense SNPs in the RI strains; we used these to generate strain-specific miRNA sequences. To generate counts that do not suffer from issues of multi-mapped reads, we performed two levels of collapsing. First, identical mature miRNA sequences were collapsed before mapping. Second, families of mature sequences with the same miRBase MIMAT ID were collapsed after mapping such that reads mapping to more than one member of a family were counted only once for the family. These families typically consist of multiple annotations of the same mature miRNA with slightly different endpoints.

In addition to the 59 LXS strains, we also obtained miRNA reads for the parental strains ILS (3 replicates) and ISS (4 replicates). 411 samples were sequenced in 5 batches. Four samples were sequenced in multiple batches to serve as controls.

To eliminate batch effects and possible other unwanted sources of variation, we applied an extension of the factor analysis based approach, the Remove Unwanted Variations using residuals (RUVr) method introduced in [1]. First, the miRNAs with consistently low reads were filtered out (remain miRNA each has at least 5 samples with more than 10 reads). It reduced the number of miRNAs from 1974 to 881. Post adjustment for library sizes either CPMM or NBMM was used to fit the reads per miRNA across samples. Then a principle component analysis was performed on the aggregate residuals. The leading principle components (PCs) correspond to unwanted variations. Our preprocessed data was obtained after adjusting for those leading PCs. We used the control samples to determine which PCs to include in the adjustment. Lastly, the controls and other repeated samples (low quality repeats) were consolidated; parental strains were dropped from the dataset. Figure S2 shows the work flow for the batch correction procedure. Depending on the regression model choice, the preprocess procedures are abbreviated as RUVrCPMM (or CP-proc) and RUVrNBMM (or NB-proc) for CPMM and NBMM, respectively. Note that CP-proc produces non-integers, while NB-proc gives integer values.

The Relative Log Expression (RLE) plots indicate that both NB-proc and CP-proc eliminates batch effects, as the post-adjustment boxplots are more uniform across samples. Overall, NB-proc shows slightly better adjustment than CP-proc Figure S3. Therefore, Simulation III is based on NB-proc processed data.

We performed a similar simulation for the CP-proc processed dataset as well (not shown here). Figure S4 shows the mean-variance relationships for the original LXS miRNA data and a simulated dataset based on each preprocess procedure on the log scale. The two processes produce almost identical mean-variance relationships. The plots for the simulated datasets show similar shapes. The preprocess model choice is in fact not as important as the model that the data were generated.

As for the real data, we applied the VPC estimation methods to both NB-proc and CP-proc preprocessed datasets, and cross compared the top heritable miRNAs.

1.3 LXS mRNA data sequencing, mapping and quantitation

Mice were administered normal saline (0.01 ml/g) and sacrificed 8 hours later by CO_2 inhalation followed by decapitation. Total RNA was isolated from 9 mice per strain and an equal amount of RNA from 3 mice of the same strain was pooled for each library; thus, 3 libraries per strain were prepared. Pooling in this manner reduces within-strain variance which produces an effective increase in statistical power without increasing the number of libraries [2, 3]. Samples were enriched for poly-A RNA using the Dynabeads mRNA Purification kit (Invitrogen) as directed by the manufacturer. Paired-end (2x100, expected size of 300 bp), strand-specific, cluster-ready libraries were prepared from the poly-A enriched RNA using the ScriptSeq RNA-Seq Library Preparation Kit v2 (Illumina) following the manufacturer's instructions. Sequencing was performed by the University of Colorado Denver Genomics and Microarray Core on an Illumina HiSeq 2000 Sequencing System as per the manufacturer's instructions with 6 bar-coded libraries pooled per flow-cell lane.

An "Affymetrix Mouse Diversity Genotyping Array" was used to generate the genotyping data of the LXS. This "SNP chip" is the base of analysis for generating LXS-specific alignment files for RNA sequencing data.

For each LXS strain, a genome alignment file (FASTA) and gene annotation file (gtf) were made by utilizing the genome coordinates provided by the SNP chip. The midpoint between two markers of the opposite strain were used as breakpoints for each haploblock. The ILS and ISS genome files were then combined together according to this coordinate scheme. Additionally, SNPs found within each LXS RNA seq sample identified as ILS or ISS specific near block switching events were then used to shift block coordinates if they did not agree with the current SNP chip breakpoints. In other words, if a SNP identified in the RNA sequencing does not agree with the haploblock structure defined by the SNP chip, then the block coordinates are shifted to account for the lack of SNP chip markers between recombination events.Genome version mm10 was used as base of analysis for genome coordinates. Ensemble mm10 gene annotation file was used for gtf.

Similar to the miRNA dataset, the mRNA data required adjustments to eliminate unwanted variations. After performing NB-proc and CP-proc to the mRNA dataset, we compared the RLE comparison (not shown here), and chose the NB-proc dataset.

1.4 Simulation II: More Realistic Sequencing Data

In a second set of simulations we sought to examine the behavior of the proposed methods in a more realistic setting where an entire data set of features with a wide variety of parameter combinations were simultaneously analyzed. This was done under both the NB-sim and CP-sim frameworks using a function that implemented the following algorithm:

- Necessary input parameters: number of features G, number of strains S, number of replicates per strain R_s, average total library size μ_{tls}, max dispersion value ϕ_{max}, max random effect variance σ^2_{max}
- Generate G feature specific means using the qAbundanceDist() function as described in the limma voom paper [4]. This function returns a set of proportions $\beta = (\beta_1, \cdots, \beta_G)$ for the expected total number of reads that are attributed to each feature based on the distributions of read counts in real RNA-Seq data. Then for feature g an overall mean is generated as $\alpha_g = \beta_g \cdot \mu_{tls}$
- For each feature draw a dispersion $\phi_g \sim U(0, \phi_{max})$ and a random effect variance $\sigma^2_g \sim U(0, \sigma^2_{max})$
- For CP-sim generated data only, draw $p_g \sim U(1, 2)$
- For each feature draw a set of random effects $\boldsymbol{b_g} = (b_{g1}, \cdots, b_{gS})$ where $b_{gs} \sim N(0, \sigma^2_g)$
- For each feature draw observed counts $Y_{gsr} \sim NB(\mu_{gs}, \phi_g)$ for NB-sim, or $Y_{gsr} \sim CP(\mu_{gs}, \phi_g, p_g)$ for the CP-sim version, where $\mu_{gs} = \exp(\alpha_g + b_{gs})$

The function also allows for some proportion of features to be simulated from a null model that has a heritability that is identically equal to 0 (i.e. the random effect variance $\sigma^2_g = 0$ and thus $\boldsymbol{b_g}$ is just a vector of zeros). A final option is the specification of a proportion of features that have a high heritability. This is achieved in general by forcing a small dispersion value ϕ_g and a larger random effect variance σ^2_g.

1.5 eQTL study

Using the LXS CP-proc miRNA data, we tested for the association between the miRNA features and approximately 40000 SNPs. Existing genotype data on the same 59 LXS strains were used [5]. The miRNA expressions were regressed on the strains using a compound Poisson fixed effects model and the hypothesis of no association was tested for each miRNA-SNP pair. The samples within each strain were collapsed and strain means were modeled as the response. Multiple testing adjustment was performed using FDR threshold 0.05.

2 Supplementary Results

2.1 LXS miRNA

For the NB-proc miRNA dataset, we also compared the rank–p-value correlation for the four methods (Figure S9). VST, NB-fit and CP-fit show high correlations between the ranks of VPC and p-value rankings ($\rho = 0.979, 0.9787, 0.9793$, respectively). The correlation for voom is only at 0.8081. For VST and voom, there are more features with tied ranking in adjusted p-values. Combined with VPC score estimation results, we conclude that the voom method is least preferable.

All the analysis performed for the NB-proc dataset were repeated for the CP-proc data. We observed similar results (Figure S10). The methods NB-fit, CP-fit, and VST produce highly correlated results. It is worth noting that NB-fit and VST are slightly more robust to the preprocess procedure than CP-fit. While the ranks and scores might differ between the different processed datasets, the most heritable miRNAs appear to be the same across. Their corresponding sequencing read plots can be found in Figures S11.

2.2 LXS mRNA

Based on NB-fit estimates, the following 16 genes present highest levels of heritability:

mRNA	VPC	p-value
Gm7672	0.996	2.3e-28
Apoa2	0.987	8.6e-21
Epx	0.986	1.8e-22
Lvrn	0.968	6.8e-31
Lrrc48	0.966	1.6e-44
D030028A08Rik	0.963	1.8e-44
A830036E02Rik	0.962	1.2e-49
Gm21967	0.959	4.4e-47
Krt12	0.958	2.0e-35
Gm15169	0.955	4.2e-25
Zfp429	0.955	5.6e-24
Ceacam2	0.954	2.6e-33
Gm9796	0.953	1.5e-23
Vmn2r29	0.952	8.0e-29
Ccl28	0.952	7.4e-39
Ccl27a	0.951	2.3e-39

Table S1 Top heritable LXS mRNA features under NB-fit. The corresponding p-values for testing the presence of heritability are listed in the adjacent column.

Intersecting the top 30 heritable genes derived under NB, CP, and VST, we obtained the following 8 genes.

In the main manuscript, we showed the read distribution for gene Gm5148. The other 7 genes that consistently present high VPC scores across methods (NB-fit, CP-fit, and VST) also have bimodal read distributions (Figures S12).

mRNA	VPC (NB-fit)	p-value	VPC (CP-fit)	p-value	VPC (VST)	p-value
D030028A08Rik	0.963	1.8e-44	0.980	1.3e-42	0.960	4.7e-44
A830036E02Rik	0.962	1.2e-49	0.994	1.2e-49	0.976	1.0e-52
Gm21967	0.959	4.4e-47	0.973	5.7e-47	0.965	9.0e-47
Krt12	0.958	1.9e-35	0.965	5.2e-35	0.934	8.4e-36
Ccl28	0.952	7.4e-39	0.993	3.6e-40	0.957	2.0e-43
Gm5148	0.947	5.3e-35	0.994	2.3e-33	0.939	1.4e-37
Exoc3	0.942	3.2e-44	0.964	2.4e-45	0.962	4.8e-45

Table S2 Estimated heritability scores for the features that are highly heritable across methods. The corresponding p-values for testing the presence of heritability are listed in the adjacent columns. The mRNA features are sorted according to the heritability estimates under the NB-fit method.

3 Supplementary Figures

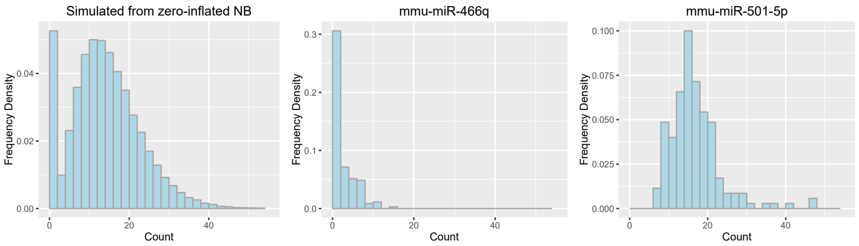

Figure S1 The left most panel shows a typical example of a zero-inflated negative binomial distribution (simulated data for illustration). We do not observe such bimodal distribution in our data. The other two panels show examples of count distribution observed in our data, either a low mean with many zeros observed (second panel) or a large mean, without many zeros observed (third panel).

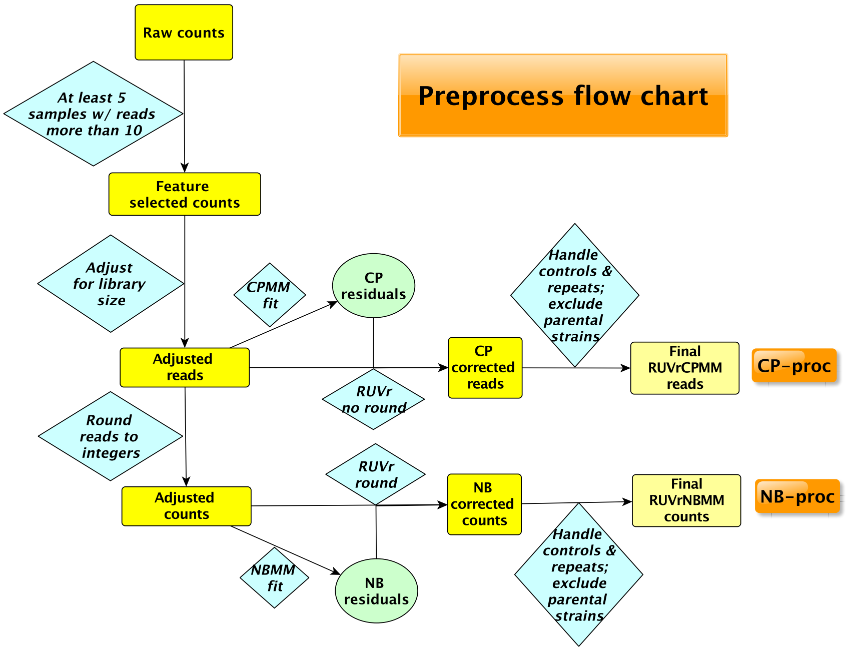

Figure S2 LXS miRNA dataset preprocess flow chart The raw sequenced data were first filtered to exclude miRNAs with consistently low reads and then adjusted for effective library sizes. Depending on choices between CP-proc and NB-proc, the adjusted reads have noise removed under either a CPMM or NBMM based model, respectively. The repeated measurements and parental strains were dropped in the last step. CP-proc produces reads on a continuous scale, while NB-proc creates integer reads (counts) at the end.

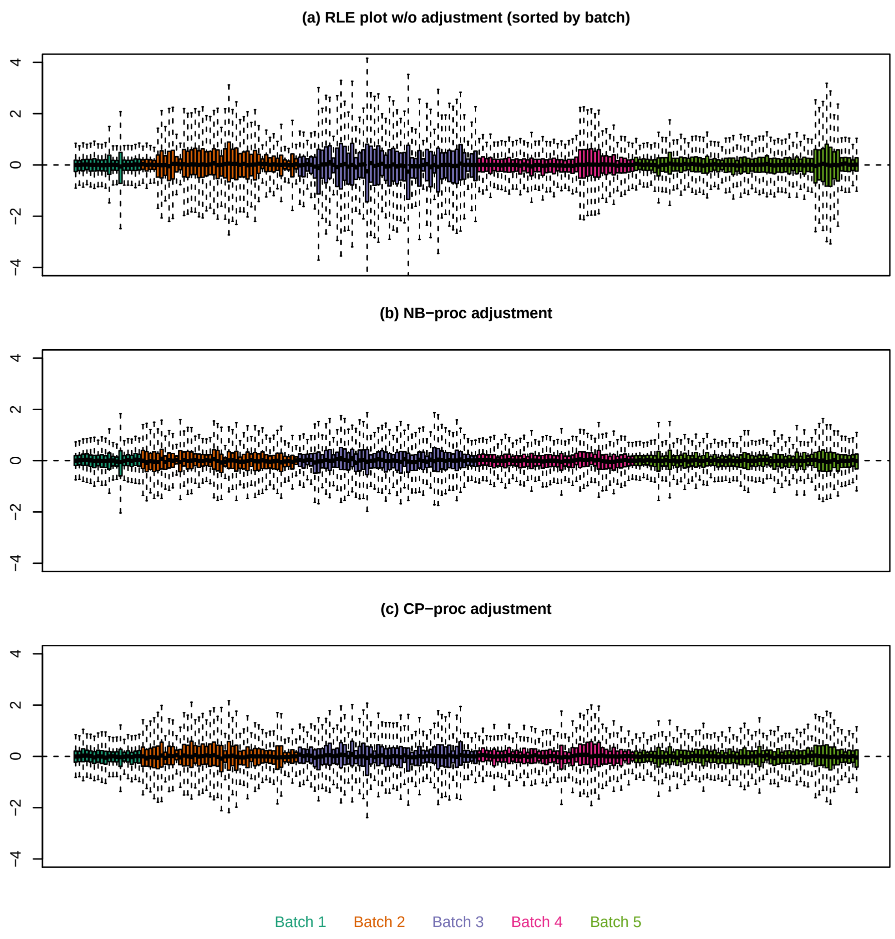

Figure S3 RLE comparison before and after adjustments. Panel (a) is for the data after feature filtering and library size adjustment. Each boxplot is one sample. The colors denote sequencing batches. Panels (b) & (c) are post NB-proc and CP-proc adjustments, respectively.

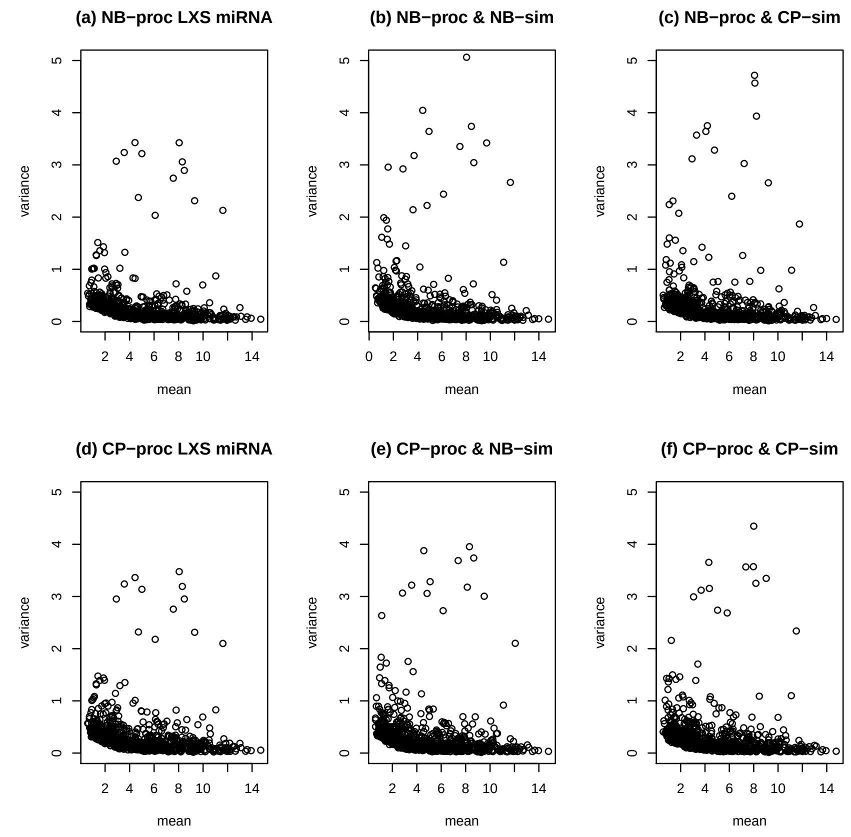

Figure S4 Mean-variance relationship of the LXS reads and simulated datasets post log-transformation. Panels (a) & (d) are the preprocessed LXS dataset, either with NB-proc or CP-proc. The four panels on the right are from simulated datasets based on either NB-sim ((b) & (e)) or CP-sim ((c) & (f)).

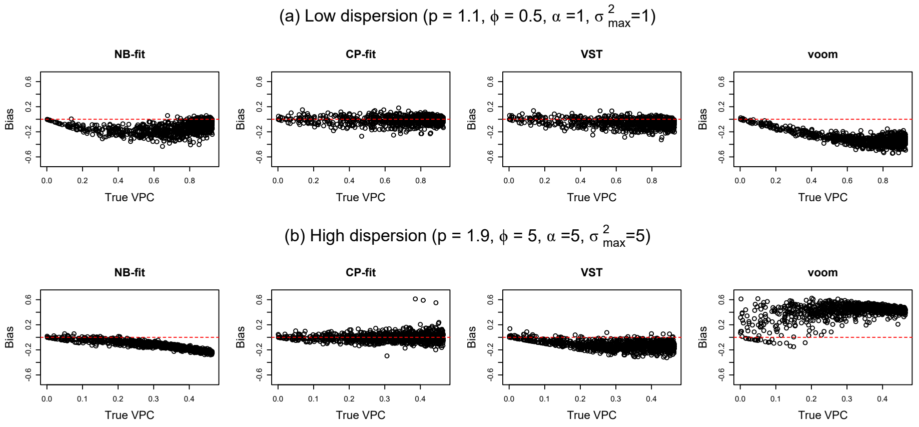

Figure S5 Comparison of bias plots for different methods estimating VPCs when the data is generated from CP-sim with different levels of dispersion. Bias is measured as estimated VPC − true VPC. (a) shows a scenario with low dispersion and (b) shows that with high dispersion.

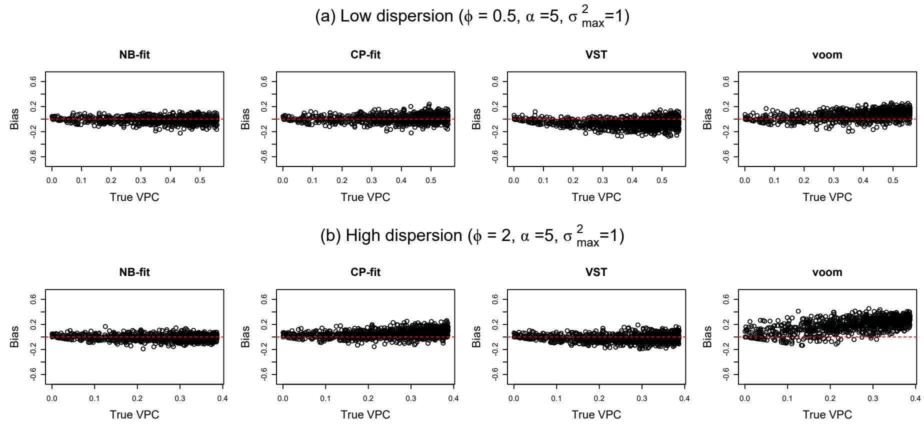

Figure S6 Comparison of bias plots for different methods estimating VPCs when the data is generated from NB-sim with different levels of dispersion. Bias is measured as estimated VPC − true VPC. (a) shows a scenario with low dispersion and (b) shows that with high dispersion.

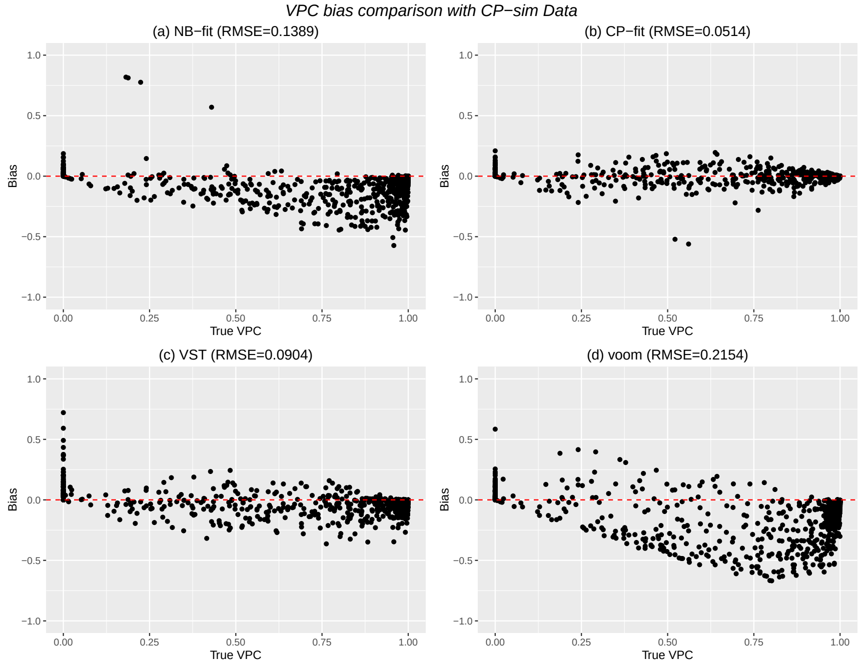

Figure S7 Bias comparisons for the four methods for a single CP-sim dataset (Simulation II) with 1000 features, 50 strains, and 3 replicates/strain. Bias is measured as estimated VPC − true VPC.

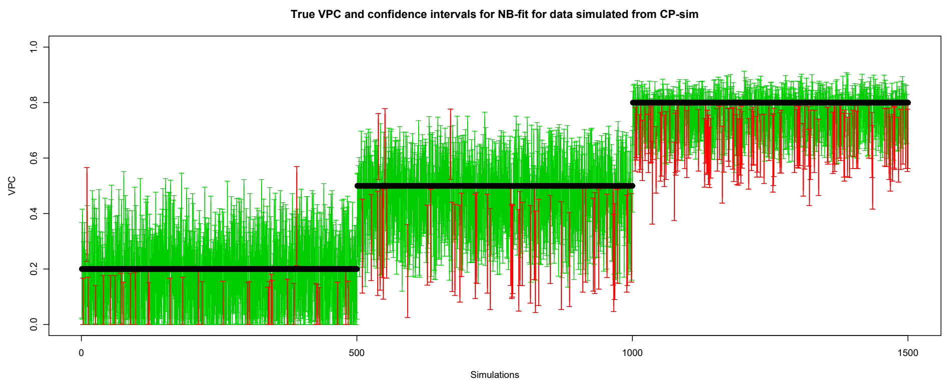

Figure S8 The confidence intervals using NB-fit when the data are generated from CP-sim. The black dots are the true values (low, medium and high), the lines represent the confidence intervals, green when an interval covers the true value and red when it does not.

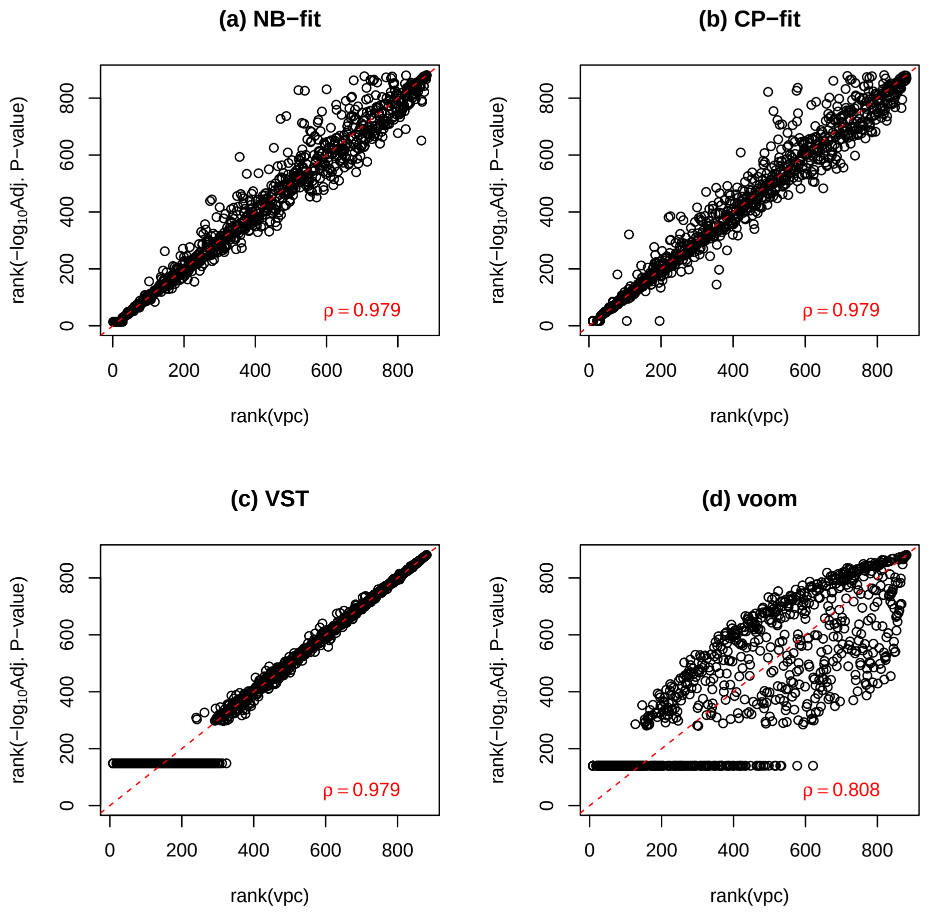

Figure S9 Scatter plot of ranks of heritability estimates and inverse log transformed p-values for the NB-proc miRNA data. The p-values have been adjusted using the Benjamini & Hochberg method. The red dashed lines show the $y = x$ line and the Spearman correlations are at the bottom right of each panel.

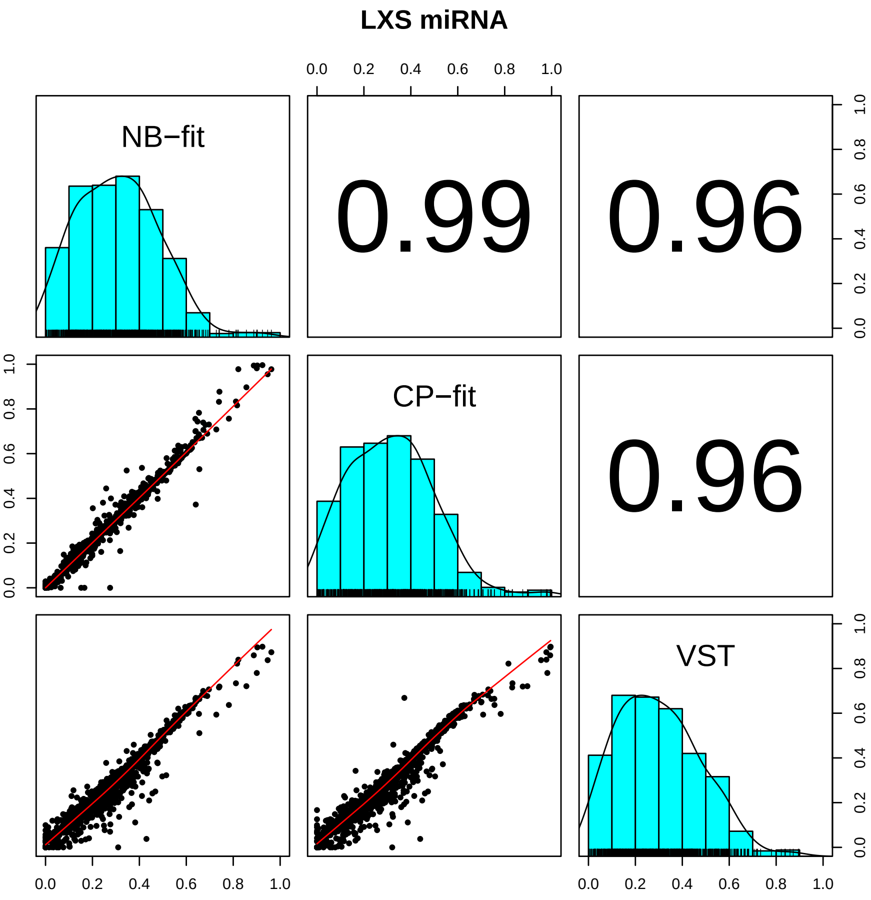

Figure S10 Heritability estimates comparison for the CP-proc LXS miRNA dataset. The histograms with rugs (ticks along the x-axis) and kernel density plots are shown along the diagonal. The panels below the diagonal show the scatter plots and the LOESS fits for the pairwise comparisons. The corresponding correlation values are listed above the diagonal.

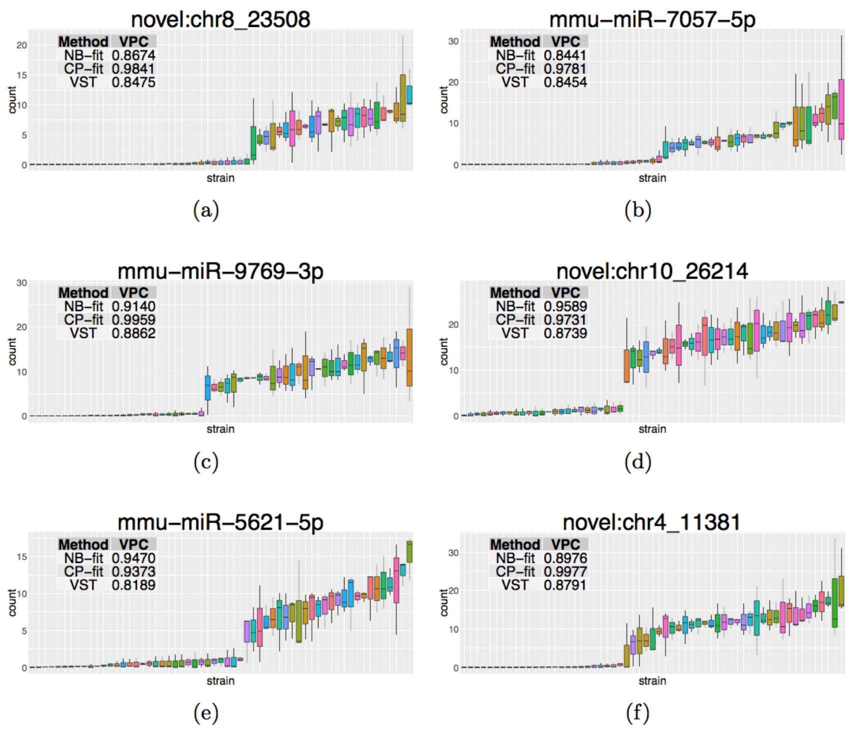

Figure S11 Sequence reads and heritability estimates for the top heritable LXS miRNAs: (a) novel:chr8_23508, (b) mmu-miR-7057-5p, (c) mmu-miR-9769-3p, (d) novel:chr10_26214, (e) mmu-miR-5621-5p, and (f) novel:chr4_11381. The estimated VPC scores are based on the NB-proc dataset.

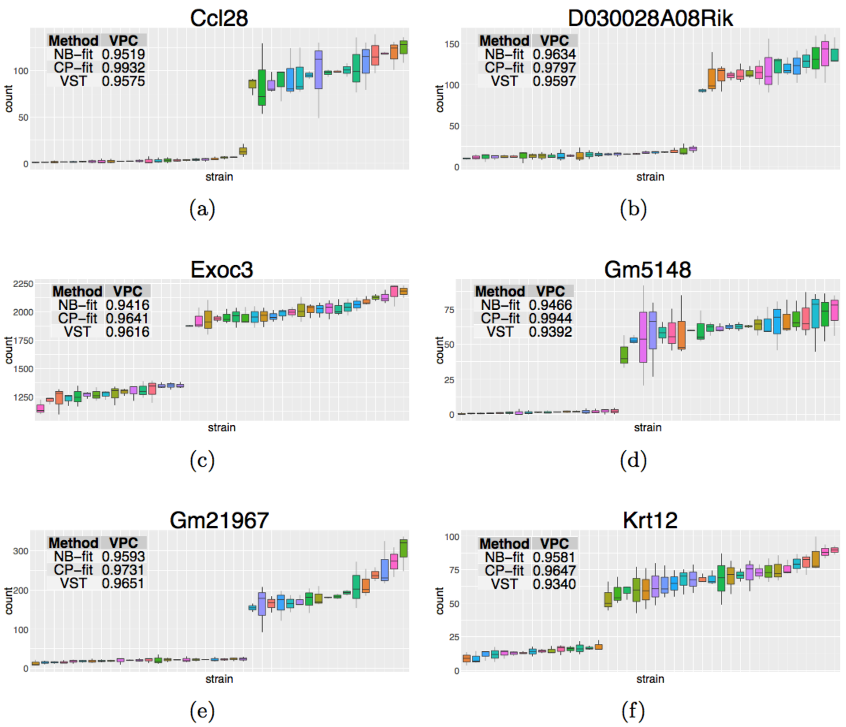

Figure S12 Sequence reads and heritability estimates for the top heritable LXS genes across methods: (a) Ccl28, (b) D030028A08Rik, (c) Exoc3, (d) Gm5148, (e) Gm21967, and (f) Krt12. The estimated VPC scores are based on the NB-proc dataset.

Author details
[1]Department of Biostatistics and Informatics, University of Colorado School of Public Health, CO 80045 Aurora, USA. [2]Computational Bioscience Program, University of Colorado School of Medicine, CO 80045 Aurora, USA. [3]Department of Biology, University of Oregon, OR Eugene, USA. [4]Department of Molecular, Cellular and Developmental Biology, University of Colorado at Boulder, CO 80309 Boulder, USA. [5]BioFrontiers Institute, CO 80303 Boulder, USA. [6]Department of Pharmaceutical Sciences, University of Colorado Skaggs School of Pharmaceutical Sciences, CO 80045 Aurora, USA.

References

1. Risso, D., Ngai, J., Speed, T.P., Dudoit, S.: Normalization of rna-seq data using factor analysis of control genes or samples. Nature biotechnology **32**(9), 896–902 (2014)
2. Kendziorski, C., Irizarry, R., Chen, K.-S., Haag, J., Gould, M.: On the utility of pooling biological samples in microarray experiments. Proceedings of the National Academy of Sciences of the United States of America **102**(12), 4252–4257 (2005)
3. Kendziorski, C., Wang, P.: A review of statistical methods for expression quantitative trait loci mapping. Mammalian genome **17**(6), 509–517 (2006)
4. Law, C.W., Chen, Y., Shi, W., Smyth, G.K.: Voom: precision weights unlock linear model analysis tools for rna-seq read counts. Genome Biol **15**(2), 29 (2014)
5. Yang et al., H.: Subspecific origin and haplotype diversity in the laboratory mouse. Nature genetics **43**(7), 648–655 (2011)
